# Cecal Volvulus a Rare Cause of Intestinal Obstruction. A Case Report

**DOI:** 10.7759/cureus.30560

**Published:** 2022-10-21

**Authors:** Abbas A Mohamed, Mohammed Alharbi, Ibrahim Alrashidi, Sarah Mohamed

**Affiliations:** 1 Surgery, King Salman Specialized Hospital, Hail, SAU; 2 Surgery, Imam Mohammad Ibn Saud Islamic University, Riyadh, SAU; 3 Medicine, University of Hail College of Medicine, Hail, SAU; 4 Surgery, University Hospital of Wales, Cardiff, GBR

**Keywords:** right hemicolectomy, cecopexy, ct -scan, intestinal obstruction, cecal volvulus

## Abstract

Cecal volvulus (CV) is a rare cause of acute intestinal obstruction caused by torsion or twisting of a mobile cecum and ascending colon. Early diagnosis and management are essential to prevent serious complications such as bowel gangrene, cecal perforation, and generalized peritonitis. We report a case of cecal volvulus with impending cecal perforation.

## Introduction

Caecal volvulus (CV) is a rare cause of intestinal obstruction, defined by an axial torsion of the cecum, ascending colon, and terminal ileum around the mesenteric vascular pedicles. [1] The incidence of caecal volvulus is 2.8-7.1 cases per million annually, and it causes approximately 1-1.5% of all intestinal obstructions [2]. Due to its infrequent occurrence and nonspecific clinical symptoms and signs, a definitive diagnosis of cecal volvulus is often delayed. We present a 53-year-old female patient who presented with intestinal obstruction and proved on a CT scan to have cecal volvulus.

## Case presentation

A 54-year-old female patient presented at the emergency room in our hospital with a history 0f three days of acute upper abdominal pain. The pain was sudden onset, colicky in nature, off and on, associated with frequent vomiting of bile-stained vomitus, abdominal distension, and absolute constipation. She went to another hospital two days before her presentation to our emergency where she was diagnosed with constipation and fecal impaction and was discharged on an oral laxative. She is a known diabetic on insulin and hypertensive, using oral Amlodipine 5mg and Lisinoprillinsopril 10mg daily. She had a hysterectomy five years ago and a laparoscopic cholecystectomy ten years ago. On examination, her weight was 92 Kg, BMI was 38. She looks ill, in pain, and moderately dehydrated. She was afebrile with a pulse of 110/minute and; a BP of 100/60 mm of Hg. On abdominal examination, there was distention in the upper right abdomen with mark tenderness over the distended area. The rest of the abdominal was soft, lax, and not tender. Bowel sounds were absent. Rectal examination revealed an empty rectum vault.

**Figure 1:**
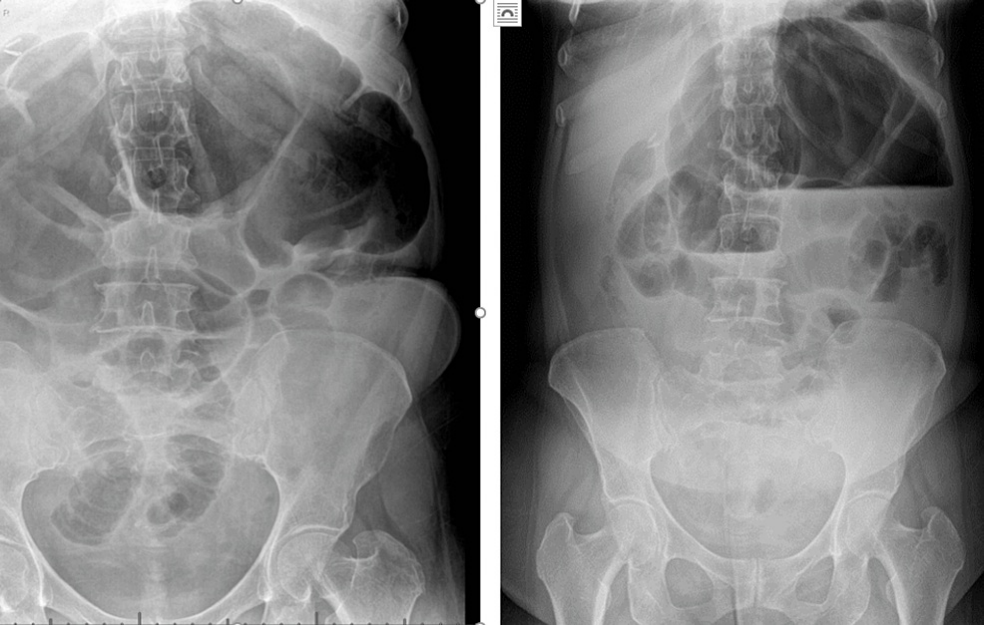
Plain X-RAY of the erect abdomen (on the left ) showing distended loops of the large bowel extending across the upper abdomen, and supine (on the right) showing fluid levels.

Laparotomy investigations showed hemoglobin of 11.3 gram%. White blood cell count OF 14.6/mm3, liver function test, urea and electrolytes, and coagulation screen within normal limits. The plain ray showed distended loops of the large bowel, extending across the upper abdomen with multiple air-fluid levels suggestive of large bowel obstruction (figure 1).

**Figure 2:**
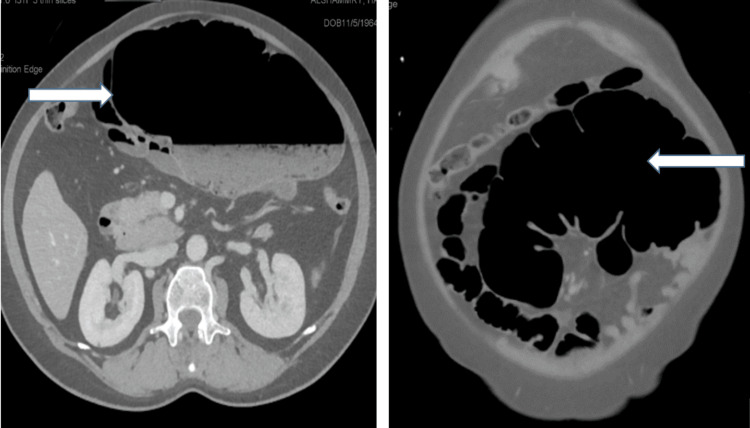
Tow (axial views) of the CT scan abdomen showing marked distension of the cecum measuring about 12 mm and extending from the right lower quadrant to the epigastrium (white arrows).

**Figure 3:**
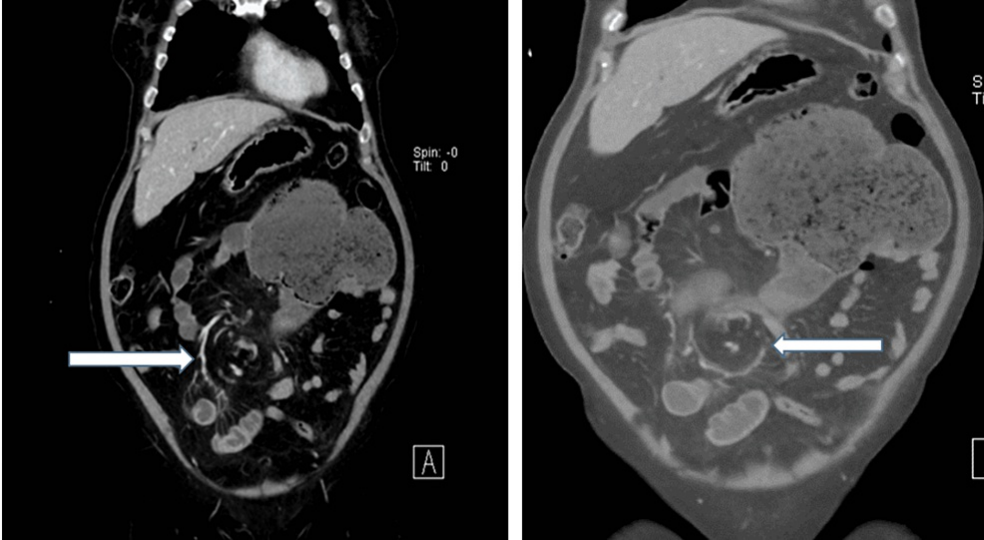
Two (coronal view) of the CT abdomen showing the whirl Sign (white arrows).

She had an abdominal CT scan which reported marked distension of the cecum measuring about 12 Cm, extending from the right lower quadrant to the epigastrium and freely mobile. There is a twist of the mesentery and mesenteric vessels (Whirl Sign). Mural enhancement suggests impending ischemic changes (Figure 2, 3). Minimal pelvic collection and no discreet mass lesion or bowel perforation. The rest of the bowel Loops look normal. The CT finding match with cecal volvulus with impending vascular compromise.-perforation.

**Figure 4:**
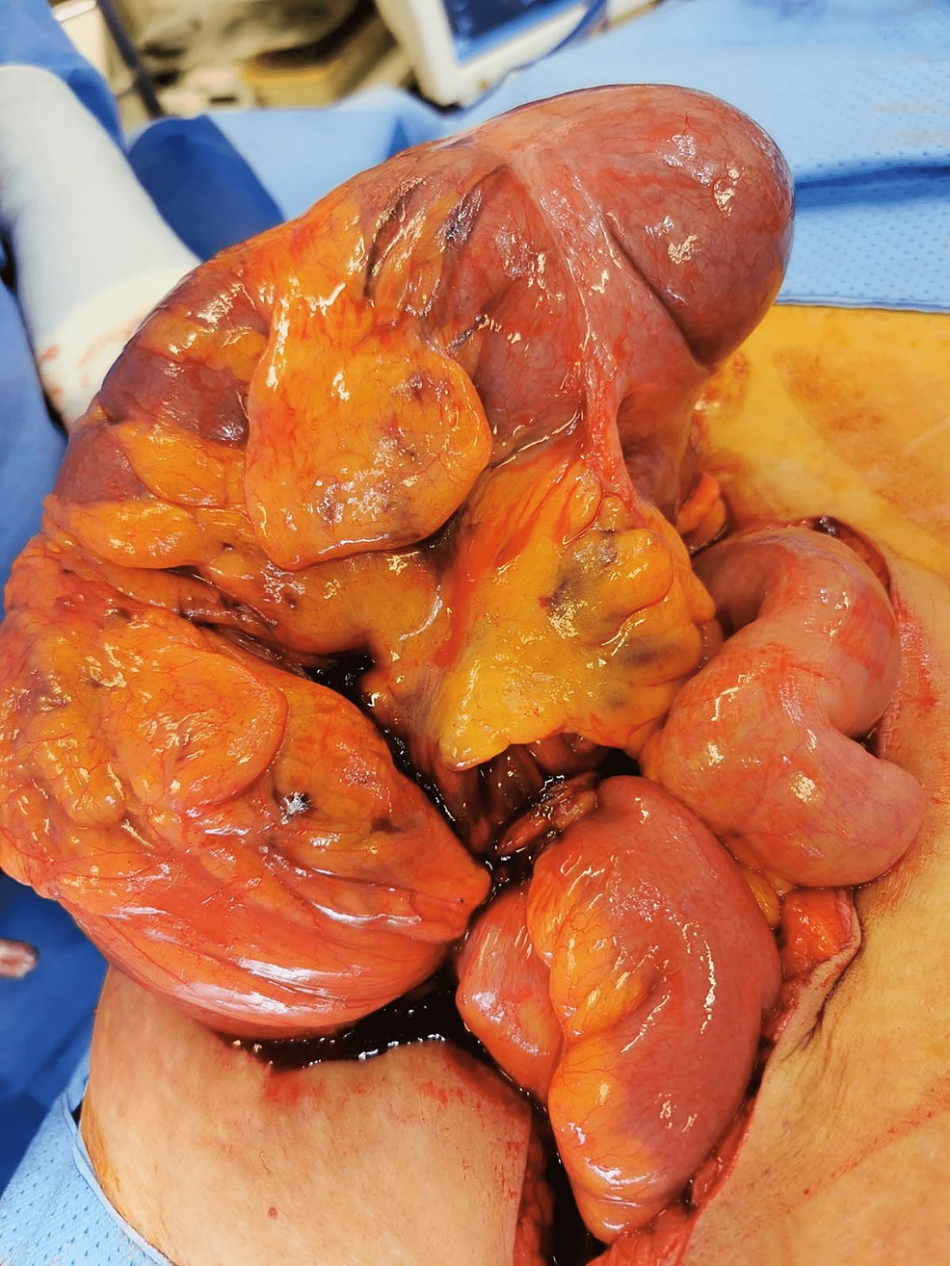
An intra-operative photograph showing the twisted distended cecum.

**Figure 5:**
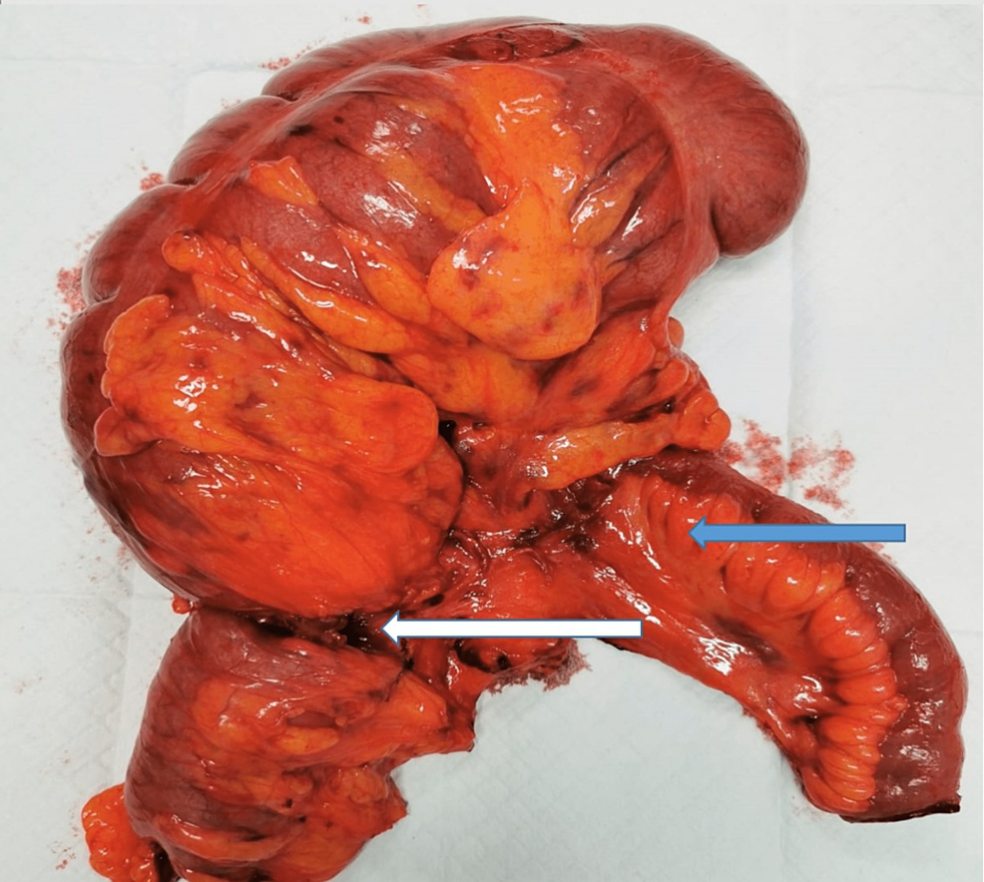
The resected specimen showing the terminal ileum (blue arrow) and the twist area (white arrow).

The patient had an emergency exploratory laparotomy through a mid-line incision. The caecum was found twisted, grossly distended, and lying in the upper right abdomen. Because of multiple serosal tears, the decision was taken for a limited right hemicolectomy. The two ends of the bowel were primarily anastomoses using a GEA 60 charge (figure 4, 5). A suction drain was inserted into the pelvis, and the laparotomy wound was closed by a mass closure using number one PDS sutures.

The patient had an uneventful postoperative recovery. Venous thromboembolism prophylaxis was given using enoxaparin subcutaneously at a dosage of 4000 UI once daily. A liquid diet was initiated 72 hours after surgery. She was discharged on the fifth postoperative day.

## Discussion

Caecal volvulus is an axial torsion of the cecum, ascending colon, and terminal ileum around the mesentery and vascular pedicles [3]. The incidence of cecal volvulus is 2.8-7.1 cases per million annually, and it is a rare cause of intestinal obstruction, accounting for approximately 1-1.5% of all intestinal obstructions [1]. A mobile cecum that is present in 25% of the general population and caused by deficient colonic fixation to the peritoneum or colon elongation resulting from over-rotation during embryologic development is the main predisposing factor for cecal volvulus [4,5]. The Clinical symptoms and signs are exceedingly variable. However, common symptoms are acute onset of severe abdominal pain, constipation, obstipation, nausea, and vomiting associated with abdominal distension and exaggerated or silent bowel sounds. Due to its infrequent occurrence and nonspecific clinical symptoms and signs, a definitive diagnosis of cecal volvulus is often delayed.

Diagnosis can be made by plain abdominal X-ray in more than half the cases based on cecal distention (with a classical "teardrop" or "comma" appearance), proximal small bowel distention with air-fluid levels, and a grassless distal colon [5]. CT is the imaging technique of choice, not only confirming the diagnosis but also ruling out other causes of acute obstruction [6].

Signs of cecal volvulus on CT scan include cecal distention greater than 10 cm, cecal apex location, distal colon decompression, and presence of the whirl, ileocecal twist [7]. Surgery is the only treatment of cecal volvulus. Surgical procedures range from simple de-torsion to the right hemicolectomy [4]. Resection (limited or right hemicolectomy) is mandatory for gangrene and a grossly distended, thin-walled cecum. Simple de-torsion, rectopexy, and cecostomy seem less effective and more morbid options than resection and anastomosis for even viable bowel [8].

## Conclusions

Cecal volvulus is a rare cause of large bowel obstruction. Early diagnosis depends on a high index of suspicion as the symptoms and signs are unspecific. The increasing use of CT scan in patients with bowel obstruction improved early diagnosis of the condition. The main course of treatment is surgical, and modalities depend on various factors, including the general condition of the patient and the presence or absence of bowel ischemia. Bowel resection and re-anastomosis for viable bowel are more effective and less morbid than other simple procedures.
